# Draft Whole-Genome Sequence of the Green Sulfur Photosynthetic Bacterium *Chlorobaculum* sp. Strain 24CR, Isolated from the Carmel River

**DOI:** 10.1128/MRA.00116-19

**Published:** 2019-03-21

**Authors:** Shawn Freed, Sydney Robertson, Terry Meyer, John Kyndt

**Affiliations:** a College of Science and Technology, Bellevue University, Bellevue, Nebraska, USA; b Department of Chemistry and Biochemistry, the University of Arizona, Tucson, Arizona, USA; Indiana University, Bloomington

## Abstract

Green sulfur bacteria are in the family *Chlorobiaceae*, which is composed of four distinct genera, namely, *Chlorobaculum*, *Chlorobium*, *Prosthecochloris*, and *Chloroherpeton*, with *Chlorobium* species being the most commonly represented in genome studies. We have now sequenced only the fourth species of *Chlorobaculum*, which established *Chlorobaculum* sp. 24CR as a separate species and should help characterize the genus.

## ANNOUNCEMENT

The green sulfur bacteria are in the *Chlorobiaceae* family, which is an interesting group that appears to be distantly related to *Bacteroides* (1). Green sulfur bacteria are obligately anaerobic photolithoautotrophs that perform anaerobic photosynthesis with oxidation of inorganic sulfur compounds (sulfide, polysulfide, or thiosulfate) (2). They live in both fresh and saltwater habitats, but their most interesting characteristic is their tolerance for very low light conditions, where they often form tightly coupled consortia with a central motile bacterium (3, 4). All members have large, distinct light harvesting structures called chlorosomes, which contain bacteriochlorophyll proteins and carotenoids (5–7). Chlorosomes are comprised of specific proteins connected to the reaction center though the Fenna-Matthews-Olson (FMO) protein (5). Several *Chlorobiaceae* species have been sequenced since the first genome of Chlorobaculum tepidum TLS in 2002 (8–11). These genomes are relatively small (2 to 3 Mbp), which reflects the biochemical simplicity of this phylum (5, 8, 12). Because of the challenges with obligately anaerobic cultivation, *Chlorobaculum* strains are underrepresented in genome sequence studies compared with other photosynthetic organisms. Nevertheless, they play an important environmental role in the geochemical sulfur cycle in nature.

The *Chlorobaculum* sp. 24CR strain was isolated by N. Pfennig from the Carmel River (California), near Hopkins Marine Station in Monterey around 1960. A pure culture was established on standard *Chlorobium* medium with acetate and thiosulfate. We isolated DNA from decades-old frozen cells, using the GeneJET DNA purification kit (Thermo Scientific), in order to examine potential differences in this strain compared with the other sequenced members of *Chlorobiaceae*. The quantity and quality of DNA were determined using Qubit and NanoDrop instruments and showed a 260/280 ratio of 1.81. The DNA library was prepared with the Nextera DNA flex library prep kit (Illumina). The genome was sequenced using 500 μl of a 1.8-pM library with an Illumina MiniSeq instrument, using paired-end sequencing (2 × 150 bp). This sequencing generated 1,892,452 reads, yielding a total of 163.86 Mbp. Quality control of the reads was performed using FASTQC within BaseSpace (Illumina, version 1.0.0), using a kmer size of 5 and contamination filtering. The data were assembled *de novo* using the Velvet application (version 1.2.10) (13) within BaseSpace (Illumina). The assembled genome consists of 109 contigs (>500 bp), with the largest contig being 154,777 bp, and an *N*_50_ value of 62,441 bp. The G+C content was 56.7%. The genome sequence was annotated using Rapid Annotations using Subsystems Technology (RAST; version 2.0) (14), which showed the whole-genome sequencing (WGS) project to be 2,772,917 bp in length and identified 2,958 coding DNA sequences (CDSs) and 51 RNAs.

A BLAST (NCBI) comparison of the 16S rRNA subunit shows 98% identity to Chlorobaculum parvum DSM263 (1,472/1,508 bp) and 97% to Chlorobaculum tepidum TLS (1,462/1,508 bp). As expected for a green sulfur bacterium, *Chlorobaculum* sp. 24CR has a set of chlorosome genes, including A, B, C, D, E, F, H, I, J, and X, and the BchlA-containing FMO protein. It also contains the *Sox FXYZAB* genes for thiosulfate oxidation.

A JSpecies comparison (15) of the average percentage nucleotide identity (ANI) between *Chlorobaculum* sp. 24CR and other published *Chlorobaculum* genomes gave the following percentages: Chlorobaculum limnaeum DSM1677, 85.8%; Chlorobaculum tepidum TLS, 84.8%; and Chlorobaculum parvum DSM263, 81.0%. Thus, *Chlorobaculum* sp. 24CR appears to be approximately equidistant to the other three *Chlorobaculum* species that have been sequenced. They are more distant to the *Chlorobium*, *Prosthecochloris*, and *Chloroherpeton* species at about 70% identity. However, these numbers are clearly below the proposed 95% cutoff for genome definition of a species, which suggests that *Chlorobaculum* sp. 24CR should be recognized as a separate species.

### Data availability.

This whole-genome shotgun project has been deposited at DDBJ/ENA/GenBank under the accession number SDGU00000000. The version described in this paper is the first version, SDGU01000000. The raw sequencing reads have been submitted to the SRA and the corresponding accession number is SRR8483032.
